# Therapeutic and Safety Evaluation of Combined Aqueous Extracts of *Azadirachta indica* and *Khaya senegalensis* in Chickens Experimentally Infected with* Eimeria* Oocysts

**DOI:** 10.1155/2016/4692424

**Published:** 2016-02-16

**Authors:** J. G. Gotep, J. T. Tanko, G. E. Forcados, I. A. Muraina, N. Ozele, B. B. Dogonyaro, O. O. Oladipo, M. S. Makoshi, O. B. Akanbi, H. Kinjir, A. L. Samuel, T. E. Onyiche, G. O. Ochigbo, O. B. Aladelokun, H. A. Ozoani, V. Z. Viyoff, C. C. Dapuliga, A. A. Atiku, P. A. Okewole, D. Shamaki, M. S. Ahmed, C. I. Nduaka

**Affiliations:** ^1^Biochemistry Division, National Veterinary Research Institute, PMB 01, Vom, Nigeria; ^2^Parasitology Division, National Veterinary Research Institute, PMB 01, Vom, Nigeria; ^3^Virology Division, National Veterinary Research Institute, PMB 01, Vom, Nigeria; ^4^Central Diagnostics Laboratory, National Veterinary Research Institute, PMB 01, Vom, Nigeria; ^5^Haematology Department, Federal College of Veterinary and Medical Laboratory Technology, PMB 01, Vom, Nigeria; ^6^Department of Veterinary Microbiology and Parasitology, University of Maiduguri, Bama Road, Maiduguri, Borno State, Nigeria; ^7^Department of Veterinary Physiology, Pharmacology and Biochemistry, University of Ibadan, PMB 0248, Ibadan, Nigeria; ^8^Department of Biochemistry, University of Ibadan, PMB 0248, Ibadan, Nigeria; ^9^Department of Medical Laboratory Science, Rivers State University of Science and Technology, PMB 5080, Port Harcourt, Nigeria; ^10^Department of Epidemiology, University of Buea, P.O. Box 63, Buea, Cameroon; ^11^Microbiology Department, Kwame Nkrumah University of Science and Technology, Kumasi, Ghana; ^12^National Veterinary Research Institute, Vom, Nigeria; ^13^Africa Education Initiative (NEF), 9401 Sentinel Ridge, Eagleville, PA 19403, USA

## Abstract

Coccidiosis is a disease of economic importance in poultry causing morbidity and mortality. Reports show that* Azadirachta indica* and* Khaya senegalensis* have been used individually in the treatment of avian coccidiosis. We thus investigated the efficacy and safety of the combined aqueous extracts of these plants for the treatment of experimentally induced coccidiosis in broiler chickens using oocyst count, oxidative stress biomarkers, serum biochemistry, histology, and haematological parameters. The phytochemical screening revealed the presence of tannins, saponins, cardiac glycosides, and steroids in both extracts. In addition, alkaloids and flavonoids were present in* Azadirachta indica.* There was significant (*p* < 0.05) dose dependent decrease in oocyst count across the treatment groups with 400 mg/kg of the combined extract being the most efficacious dose. Immunomodulatory and erythropoietic activity was observed. There were decreased intestinal lesions and enhanced antioxidant activity across the treatment groups compared to the negative control. Administration of the combined extract did not cause damage to the liver as ALT, AST, and ALP levels were significantly reduced in the uninfected chickens treated with the extracts compared to control suggesting safety at the doses used. The combined aqueous extracts of* K. senegalensis* stem bark and* Azadirachta indica* leaves were ameliorative in chickens infected with coccidiosis.

## 1. Introduction

Coccidiosis is a major parasitic disease of poultry caused by an Apicomplexan protozoan belonging to the subclass Coccidia, family Eimeriidae, and genus* Eimeria* [1]. The disease has significant economic impact on the poultry industry causing high mortality, poor growth, decreased productivity, and high medical cost [2]. Anticoccidial drugs are commonly used to prevent and treat coccidiosis. However, indiscriminate and long-time use of anticoccidial drugs has led to the emergence of drug resistant parasites and presence of residual drugs in chicken products raising concerns about public health and food safety [3, 4]. According to Yang et al. [5], anticoccidial vaccines are an alternative means to prevent coccidiosis. However, efficacy, safety, and cost-effectiveness are still challenges for anticoccidial vaccine use in poultry [6]. Consumers and poultry farmers around the world have voiced concerns about the use of present anticoccidial agents [5]. Therefore, there is an expedient need for an alternative approach to prevent and treat avian coccidiosis necessitating an examination of the potential of natural products from plant extracts.

In recent time, various researchers have tested several plants for anticoccidial activity in chickens [4, 5, 7–10].* Azadirachta indica (AI)* and* Khaya senegalensis (KS)* both belonging to the family Meliaceae have been reported to possess anticoccidial properties and have been used individually to combat avian coccidiosis. This property has been demonstrated by their ability to reduce oocyst count [11, 12], inhibit inflammation [13], and enhance erythropoiesis [14, 15]. In addition,* KS* has antidiarrhoeal properties [16]. The pathogenesis of coccidiosis is associated with oxidative stress caused by increased generation of reactive oxygen species due to activities of the parasite as well as the host immune system causing a depletion of antioxidant enzymes and GSH level and increased lipid peroxidation of cells in the intestinal linings and surrounding tissues.* AI* and* KS* have free radical scavenging ability as well as cellular immune-modulatory properties in mice [17] and human colorectal cancer [18].

Tipu et al. [19] showed that combinations of herbs used against coccidiosis are effective and an economical alternative for prophylactic anticoccidial medication.* AI* administered at dosages of 200, 400, 800, and 1600 mg/kg in broiler chickens for 4 days showed 800 mg/kg to be the most effective dosage [11]. In another study by our group using* KS* and* AI* as single extracts, 800 mg/kg was found to be most effective for each extract [unpublished data]. We therefore hypothesized that combining the extracts should be more effective at a lower dosage. This study was, therefore, aimed at evaluating the therapeutic efficacy of the combined aqueous extracts of leaves of* AI* and stem bark of* KS* in chickens experimentally infected with* Eimeria* oocyst using oocysts count, oxidative stress markers, intestinal histopathology, and haematological parameters and also determining the safety of the doses used on serum levels of liver and kidney function parameters and biomarkers of oxidative stress in liver homogenates of uninfected chickens treated with the extracts.

## 2. Materials and Methods

### 2.1. Plant Collection and Preparation

The stem bark of* KS* and leaves of* AI* were collected from the environs of the National Veterinary Research Institute (NVRI), Vom, Nigeria. The plants were identified and authenticated in the herbarium at Federal College of Forestry, Jos, Nigeria, and assigned voucher numbers FHJ 198 and FHJ 199 for* AI* and* KS*, respectively. After collection, the samples were washed, air-dried, and ground into powder under aseptic conditions. Eight hundred grams (800 g) of the individual pulverized plant was macerated with distilled water for 72 hours. At the end of the extraction, the mixture was sieved and filtered. The filtrate was concentrated by drying in the oven at 40°C [20] with modifications. The dried extracts were stored at 4°C until needed.

### 2.2. Phytochemical Screening

The extracts of both* KS* and* AI* were screened individually to detect the presence of some phytochemicals according to the methods described [21].

### 2.3. Source of Oocyst

Mixed* Eimeria* oocyst suspension (*Eimeria tenella*,* E. necatrix*, and* E. brunetti*) was obtained from the Parasitology Division of the National Veterinary Research Institute (NVRI), and each 1 mL of the oocyst suspension contained a total of 2185 mixed* Eimeria* oocysts which was determined by the Mac Master technique [22].

### 2.4. Experimental Animals

Apparently healthy day-old broiler chicks were obtained from a hatchery in Jos, Nigeria, and brooded under standard conditions for three weeks before commencement of the study. The chicks were fed standard pelletized broiler starter feed (Vital Feed® Grand Cereals, Nigeria, Plc., Jos, Nigeria) and water* ad libitum*. Birds were housed in individual cages with proper lighting and heat. The birds were vaccinated against infectious bursal disease (IBD) and Newcastle disease virus with IBD and La Sota vaccines, respectively, using NVRI, Vom, vaccines. All experiments were conducted in accordance with the Principles and Guide for the Care and Use of Laboratory Animals [23] and approved by the Animal Ethics Committee of NVRI, Vom.

### 2.5. Experimental Design

#### 2.5.1. Efficacy Study

Twenty-five (25) experimental birds were weighed and each infected with 2185 sporulated oocysts (0.1 mL) as a single oral gavage according to Biu et al. [11]. Daily collection and screening of faeces for oocyst presence and count were carried out. Birds were also monitored for clinical signs of coccidiosis. After establishment of the infection (7 days after inoculation), treatment commenced by oral gavage of the extract. The combined aqueous extracts of* AI* and* KS* were administered at a dose ratio of 1 : 1 at 100 mg/kg, 200 mg/kg, and 400 mg/kg. The experiment included two control groups, negative and positive, treated with distilled water and amprolium (Amprolium 250 WSP, Kepro® B.V., Holland), respectively. All treatments lasted for five (5) days which is the usual period of chemotherapy for coccidiosis using the standard drug amprolium. At the end of the experiment, chicks were sacrificed by cervical dislocation and blood was collected from the jugular vein into EDTA containers for haematological analysis and processed immediately. Tissues were collected in 10% buffered formalin and physiological saline solution for histopathological and oxidative stress examinations, respectively.

#### 2.5.2. Safety Study

Twenty (20) apparently healthy birds were divided into four groups of five (5) birds each. The combined aqueous extracts of* AI* and* KS* were administered at the dose ratio of 1 : 1 at 100 mg/kg, 200 mg/kg, and 400 mg/kg for 5 days while the control group received distilled water. The experiment was terminated 24 hours after the last administration. Blood was collected for serum biochemical analysis and liver harvested for oxidative stress assays.

### 2.6. Oocyst Estimation

Evaluation of faeces for the oocyst per gram (OPG) counts was performed using modified McMaster's technique [22].

### 2.7. Tissue Homogenization

The harvested tissues (intestine for therapeutic efficacy and liver for the safety study) were rinsed with phosphate buffered saline (PBS) and blotted with filter paper and weighed. They were then chopped into bits and homogenized in ice using homogenizing buffer (0.1 M phosphate buffer, pH 7.4) at ratio of 1 : 4 w/v. The resulting homogenate was centrifuged at 10,000 g for 15 minutes at −4°C to obtain the postmitochondrial fraction. The supernatant was collected, stored at −4°C, and used for oxidative stress assays. All samples were analyzed within 7 days after termination of experiment.

### 2.8. Oxidative Stress Assays

Superoxide dismutase (SOD), reduced glutathione (GSH) levels, malondialdehyde (MDA), catalase, and total protein were determined [24].

### 2.9. Haematological Evaluation

Red blood cell (RBC), white blood cell (WBC), packed cell volume (PCV), haemoglobin concentration (Hb), mean corpuscular volume (MCV), mean corpuscular haemoglobin (MCH), and mean corpuscular haemoglobin concentration (MCHC) together with absolute count of heterophils and lymphocytes as well as H/L ratio were determined [25].

### 2.10. Serum Biochemical Analysis

Alkaline phosphatase (ALP), alanine aminotransferase (ALT) and aspartate aminotransferase (AST), blood urea nitrogen (BUN), serum creatinine (CRE), total protein (TP), albumin (ALB), total bilirubin (TBIL), and direct bilirubin (DBIL) were analysed using Randox Diagnostic Test kits according to manufacturer's instructions.

### 2.11. Histopathological Examination

Tissue sample (intestine) was harvested from the infected and treated birds immediately after sacrifice, fixed in 10% buffered formal saline, embedded in paraffin wax, sectioned at 5 *μ* thickness, stained with haemotoxylin and eosin (H&E) stain, cleared in xylene, and mounted in a mountant [26, 27].

### 2.12. Statistical Analysis

Data obtained from the study were summarized as means ± standard error of mean and differences between the means determined at 5% level of significance using the one-way analysis of variance [28].

## 3. Results

### 3.1. Extraction and Phytochemical Screening

The yield of the plants was 4.90% for* AI* and 7.02% for* KS*. The phytochemical screening revealed the presence of tannins, saponins, cardiac glycosides, and steroids in both extracts. Alkaloids and flavonoids were present in* AI* while resins, terpenes, and anthraquinones were not detected in both extracts.

### 3.2. Effect of Combined Extracts on Oocyst Count and Weight Gain (Efficacy Study)

**Figure 1:**
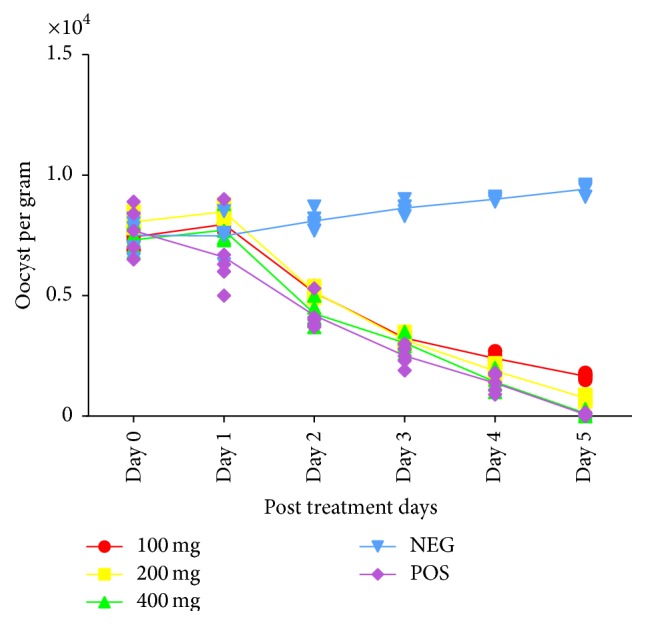
Therapeutic effect of combined extract of* Azadirachta indica* and* Khaya senegalensis* on oocyst count of chicken infected with* Eimeria* oocysts. NEG = negative control (infected, not treated). POS = positive control (infected and treated with amprolium).

**Figure 2:**
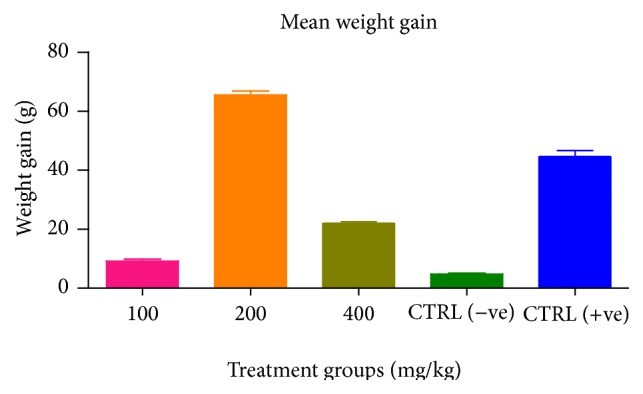
Effect of the combined extracts on weight gain of chickens infected with* Eimeria* oocysts.

A dose dependent reduction in oocyst count was observed in birds treated with the extracts (Figure 1). A significant (*p* < 0.05) increase in weight gain was also recorded among treated groups (Figure 2).

### 3.3. Effect of Combined Extracts on Haematological Parameters (Efficacy Study)

There was no significant difference (*p* > 0.05) in RBC count and haemoglobin concentration within treatment groups but an increase in mean RBC count was observed when compared with the negative control. There was a significant (*p* < 0.05) decrease in WBC count in the treated groups compared to negative control. There was no significant (*p* > 0.05) change in heterophils between the negative control and the treated groups but a dose dependent decrease in heterophils was observed (Table 1).

### 3.4. Effect of Combined Extract on Oxidative Stress Markers in the Intestine (Efficacy Study)

**Figure 3:**
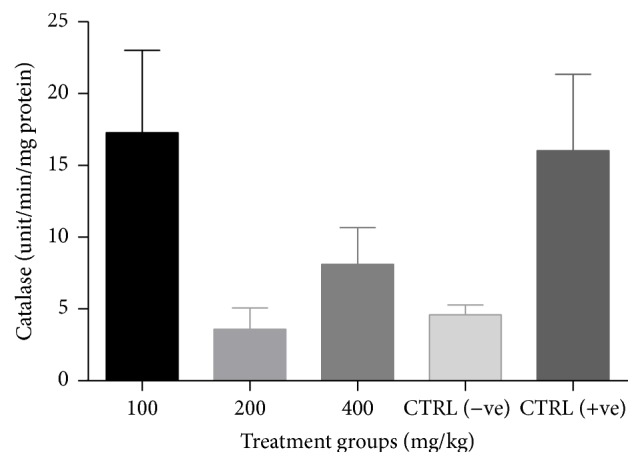
Effect of combined extracts of* Azadirachta indica* and* Khaya senegalensis* on catalase activity in the intestine of chickens infected with* Eimeria* oocysts.

**Figure 4:**
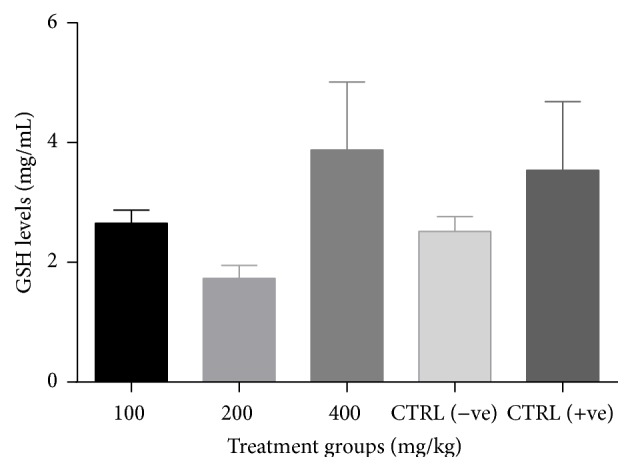
Effect of combined extracts of* Azadirachta indica* and* Khaya senegalensis* on glutathione (GSH) levels of the intestine of chickens infected with* Eimeria* oocysts.

**Figure 5:**
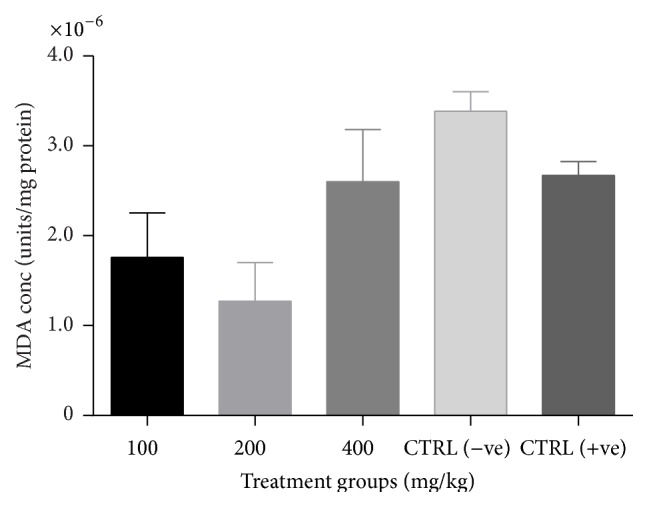
Effect of combined extracts of* Azadirachta indica* and* Khaya senegalensis* on lipid peroxidation (malondialdehyde, MDA) of chickens infected with* Eimeria* oocysts.

**Figure 6:**
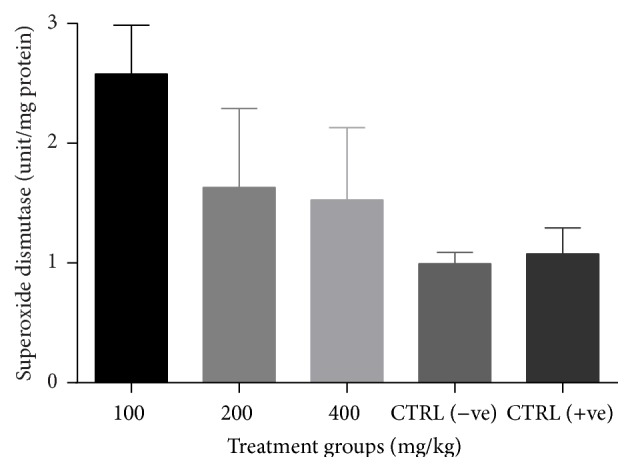
Effect of combined extracts of* Azadirachta indica* and* Khaya senegalensis* on superoxide dismutase (SOD) activity of the intestine of chickens infected with* Eimeria* oocysts.

There was a significant increase (*p* < 0.05) in catalase activity of all groups treated with the combined extract when compared to the negative control (Figure 3). No significant change (*p* > 0.05) in GSH levels (Figure 4) and SOD activity (Figure 5) was recorded between the negative control and the treated groups. However, there was a general decrease in MDA levels in all treatment groups with significant reduction (*p* < 0.05) observed at lower doses of the combined extract (Figure 6).

### 3.5. Histological Findings from the Chicken Intestine Infected with Eimeria Oocyst (Efficacy Study)

**Figure 7:**
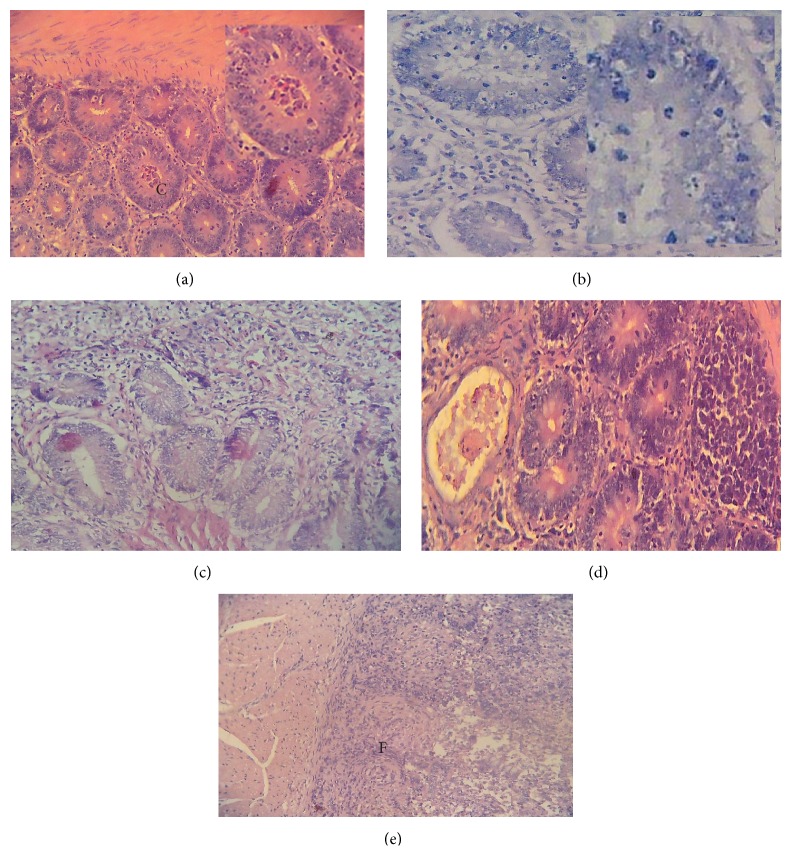
Photomicrograph of the intestine of chickens infected with* Eimeria* oocysts and treated with graded doses of the combined extract and control. (a) 100 mg/kg: combined extract of* KS* and* AI* treatment; moderate cryptic destruction and intracryptic developmental stages of* Eimeria* H&E: ×400. (b) 200 mg/kg: intestine infected with* Eimeria* spp.; moderate cryptic destruction and intracryptic developmental stages of* Eimeria* and intestinal fibrosis H&E: ×400. (c) Amprolium 10 mg/L: chicken, intestine, moderate cryptic destruction and ectasia, intracryptic developmental stages of* Eimeria*, and intestinal fibrosis H&E: ×400. (d) 400 mg/kg extract: moderate cryptic destruction and intracryptic developmental stages of Eimeria H&E: ×: 400. (e) Control (−ve) intestine infected with* Eimeria* spp.; severe cryptic destruction and intracryptic developmental stages of* Eimeria* and intestinal fibrosis H&E: ×400.

For the 100 mg/kg and 200 mg/kg of the combined extract, moderate cryptic destruction as well as intracryptic developmental stages was observed (Figures 7(a) and 7(b)). The architecture of the intestine was moderately preserved in the 400 mg/kg treated group. The crypts and the lamina propria were preserved (Figure 7(c)) similar to the positive control group (Figure 7(d)). Intracryptic developmental stages of the parasite were observed in the negative control, causing severe cryptic destruction and intestinal fibrosis (Figure 7(e)).

### 3.6. Effect of Combined Extract on the Serum Biochemical Markers of Healthy Birds (Safety Study)

Administration of the combined extract of* KS* and* AI* to apparently healthy birds significantly reduced (*p* < 0.05) ALT, AST, ALP, TP, DBIL, and TBIL across the groups treated with the extract, with the 400 mg/kg group showing a marked reduction in the levels of these serum markers compared to the control (Table 2). The albumin levels increased significantly (*p* < 0.05) across the groups treated with the extract compared to the control (Table 2).

There was a significant decrease (*p* < 0.05) in the creatinine and urea levels when compared to the control (Table 3). In addition, the combined extract did not significantly (*p* > 0.05) affect the sodium and potassium levels when compared to the control (Table 3).

### 3.7. Effect of Combined Aqueous Extract on Oxidative Stress Markers in Liver of Healthy Chickens (Safety Study)

**Figure 8:**
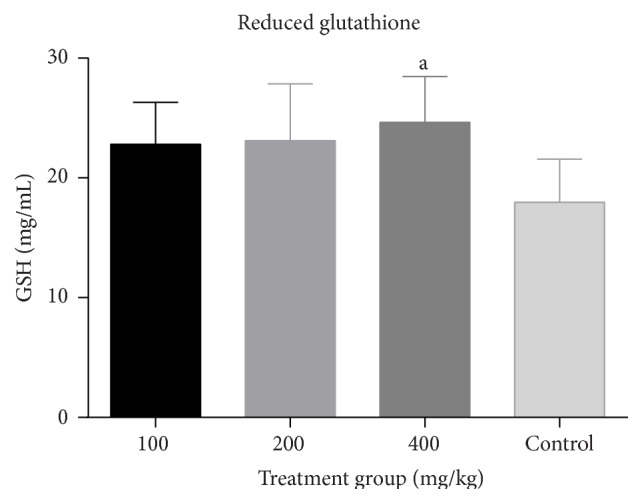
Effect of combined aqueous extracts of* Azadirachta indica* and* Khaya senegalensis* on reduced glutathione (GSH) levels of the liver of healthy chickens. Values are expressed as mean ± SEM where *n* = 5. ^a^Significant as compared with control (*p* < 0.05).

**Figure 9:**
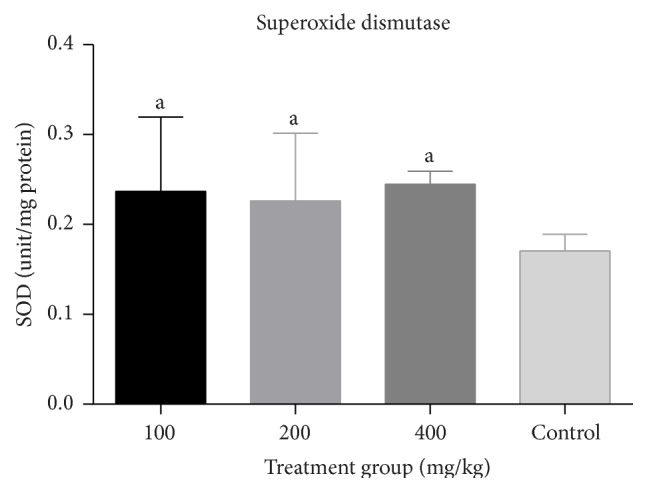
Effect of combined aqueous extracts of* Azadirachta indica* and* Khaya senegalensis* on superoxide dismutase activity (SOD) of the liver of healthy chickens. Values are expressed as mean ± SEM where *n* = 5. ^a^Significant as compared with control (*p* < 0.05).

**Figure 10:**
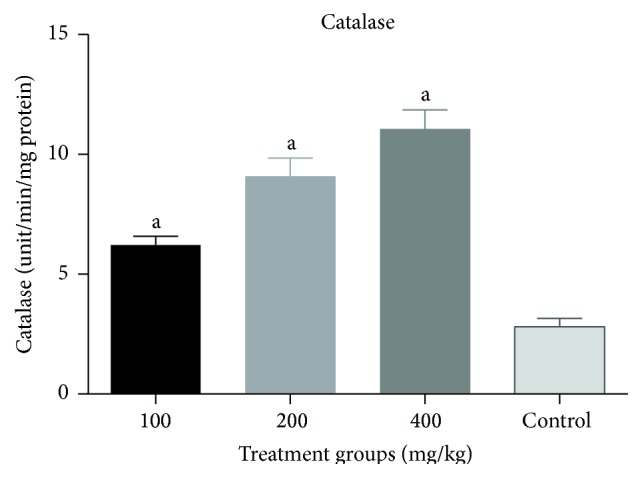
Effect of combined aqueous extracts of* Azadirachta indica* and* Khaya senegalensis* on catalase activity of the liver of healthy chickens. Values are expressed as mean ± SEM where *n* = 5. ^a^Significant as compared with control (*p* < 0.05).

There was a dose dependent increase in GSH levels which was significant (*p* < 0.05) at 400 mg/kg when compared to the control. Similarly, there was a significant dose dependent increase (*p* < 0.05) in SOD and catalase activities when compared to the control (Figures 8, 9, and 10).

## 4. Discussion

The dose dependent reduction in oocyst count observed in the treated groups was comparable with amprolium which could be attributed to the presence of some bioactive compounds in the plant extracts. Saponin, for example, is known to bind membrane cholesterol, altering the integrity of the parasite membrane, resulting in loss of homeostasis and eventual death of the parasite [29]. Also, limonoids contained in AI inhibit protein digestion and uptake of vitamins and minerals by the parasites in the gut [17]. This action results in impaired nutrient utilization, reduced growth, and multiplication of the parasite which could contribute to the reduced oocyst count observed.

Extracts of neem and mahogany when used individually have been reported to reduce oocyst count in avian coccidiosis [11, 12]. In a separate study carried out by our group, similar observation was made when each plant extract was administered singly (data not shown). However, in this study, the oocyst count was more reduced when the combined extract was used.

The significant increase in mean weight gain in treated birds when compared to the negative control is possibly due to the inhibition of inflammation in the intestinal mucosa which is suggestive of an increased nutrient absorption across the intestinal wall and enhanced feed conversion ratio compared to the negative control. Nwosu et al. [12] and Biu et al. [11] reported an increased weight gain and feed conversion ratio in birds treated with only KS extracts and Al, respectively.

The observed increase in RBC and haemoglobin concentration is indicative of the erythropoietic ability of the combined extracts, which is beneficial since the* Eimeria* parasite in the epithelia of the intestines causes bloody diarrhoea and consequently anaemia. This finding is in consonance with that of Sanni et al., [15] who reported an antianaemic effect of KS on phenylhydrazine-induced anaemia in rats.

AI has been shown to possess antianaemic properties in rats [14]. The dose dependent decrease in white blood cells count and heterophil-lymphocyte ratio with a concomitant reduction in heterophils is suggestive of decreased inflammation. It can be extrapolated that the decrease in parasitic load downregulates the activity of the immune system leading to decrease in inflammation and consequently a decrease in heterophils, tending towards the normal blood picture of a greater ratio of lymphocytes to heterophils in avian species. In addition,* Khaya senegalensis* has been reported to inhibit inflammation in rats [13].

The increase in intestinal glutathione level, catalase, and superoxide dismutase activity as well as decrease in malondialdehyde levels in the treated groups is suggestive of the* in vivo* antioxidant enhancing capacity of the combined extract. During* Eimeria* infection in chickens, the innate immune system of these chickens protects them by producing reactive oxygen species (ROS) in a process termed “oxidative burst” in an attempt to destroy the* Eimeria* pathogens [30]. Unfortunately, since such ROS generated are not target specific, the reactive species also damage cells of the gastrointestinal tract resulting in ulcers [31]. Flavonoids, limonoids, and saponins, amongst other active phytocompounds, present in the plant extracts possess antioxidant properties which aid in free radical scavenging and reactive oxygen quenching activities, thereby ameliorating oxidative stress mediated damage [13, 17, 18]. AI aqueous extract has been reported to reduce MDA levels in mice infected with* Eimeria* species [17]. In addition, KS was reported to also reduce MDA levels in rats with ethanol-induced ulcers [13].

From the result of the safety study, the reduction in AST, ALT, and ALP activities compared to control shows that the combination of the extracts is not hepatotoxic. The elevated catalase and superoxide dismutase activities in the liver of the chicken may suggest that administration of combined extracts of KS and AI could be hepatoprotective. It has been previously reported that the methanolic extract of AI leaves (500 mg/kg) and aqueous extract of KS stem bark (250 and 500 mg/kg, resp.) have hepatoprotective activity [17, 32]. The increase in albumin levels observed in the different dose levels is suggestive of proper maintenance of the integrity of the liver and other extrahepatic tissues involved in protein synthesis [33].

The significant decrease in serum creatinine and urea levels across the dose levels when compared to the control shows that the extract combination was not nephrotoxic. In addition, the sodium and potassium levels were not affected, thus giving further credence to the safety of the extracts on the kidney. Decreased urea level with no marked changes in serum potassium and sodium levels following supplementation of broiler feed with neem leaves has been reported [33].

## 5. Conclusion

This study shows novel findings with respect to the possible synergistic efficacy of the combination of aqueous extracts of* K. senegalensis* stem bark and* A. indica* leaves lending further credence to the folkloric use of these plants in the treatment of coccidiosis.

## Figures and Tables

**Table 1:** Effect of combined extracts of *Azadirachta indica* and *Khaya senegalensis* on red blood cell count, haemoglobin concentration, total white blood cell/differential counts of chickens infected with *Eimeria *oocysts.

Treatment	RBC × 10^12^	Hb (g/dL)	WBC × 10^9^	Heterophils (%)	Lymphocyte (%)
Control (−ve)	1.944 ± 0.121	11.00 ± 1.266	12.32 ± 0.476	25.80 ± 3.137	78.33 ± 2.906
Control (+ve)	2.466 ± 0.370	12.20 ± 0.938	10.44 ± 0.33^a^	23.00 ± 4.438	73.50 ± 3.524
100 mg/kg	2.123 ± 0.358	14.04 ± 0.248	9.02 ± 0.233^a^	27.80 ± 4.067	42.40 ± 3.894^a,b^
200 mg/kg	2.107 ± 0.066	12.82 ± 1.352	8.448 ± 0.354^a,b^	25.20 ± 3.527	40.20 ± 5.417^a,b^
400 mg/kg	2.040 ± 0.050	11.17 ± 0.667	10.56 ± 0.640^a^	20.00 ± 2.983	76.33 ± 3.283

Values in the same column with different letters superscripts are significantly different.

**Table 2:** Clinico-chemical parameters of liver function of healthy chickens exposed to combined aqueous extract of *Azadirachta indica *and *Khaya senegalensis*.

Treatment	Parameter						
	ALT (U/L)	AST (U/L)	ALP (U/L)	TP (g/L)	TBIL (mg/dL)	DBIL (mg/dL)	ALB (mg/dL)
Control	8.320 ± 0.489^b^	21.32 ± 0.598^b^	447.80 ± 7.055^b^	35.32 ± 2.71^b^	0.444 ± 0.005^b^	0.233 ± 0.024^b^	9.083 ± 1.124^b^
100 mg/kg	7.560 ± 0.796^b^	19.84 ± 0.379^b^	420.70 ± 3.143^a^	32.89 ± 1.459^b^	0.406 ± 0.072^b^	0.130 ± 0.017^a^	14.16 ± 1.089^a^
200 mg/kg	6.927 ± 0.058^b^	18.89 ± 0.741^a^	412.70 ± 6.374^a^	26.51 ± 0.685^a^	0.399 ± 0.032^b^	0.087 ± 0.048^a^	14.83 ± 0.724^a^
400 mg/kg	5.600 ± 0.489^a^	16.10 ± 2.330^a^	312.40 ± 4.347^a^	28.00 ± 0.907^a^	0.358 ± 0.035^a^	0.120 ± 0.032^a^	13.88 ± 0.409^a^

Values are expressed as mean ± SEM.

Columns with different superscript show significant difference (*p* < 0.05).

**Table 3:** Clinicochemical parameters of kidney function of chickens exposed to combined aqueous extract of *Azadirachta indica* and *Khaya senegalensis*.

Treatment	Parameter			
	CRE (mg/dL)	URE (mg/dL)	K (mEq/L)	Na (mEq/L)
Control	0.418 ± 0.024^b^	9.179 ± 0.540^c^	5.613 ± 0.248^a^	147.43 ± 1.003^b^
100 mg	0.288 ± 0.001^a^	5.847 ± 0.540^a^	5.013 ± 0.622^a^	150.89 ± 1.028^b^
200 mg	0.285 ± 0.039^a^	5.439 ± 0.490^a^	5.403 ± 0.315^a^	151.33 ± 4.290^b^
400 mg	0.285 ± 0.039^a^	7.207 ± 0.544^a^	5.957 ± 0.915^a^	147.17 ± 0.301^b^

Values are expressed as mean ± SEM.

Rows with different superscript show significant difference (*p* < 0.05).
